# Determination of ultrasound reference values for diagnosing low muscle mass in older Chinese adults

**DOI:** 10.1093/geroni/igaf122.259

**Published:** 2025-12-31

**Authors:** Qin Zhang, Shumin Li, Jing Chen

**Affiliations:** The First Affiliated Hospital, Zhejiang University School of Medicine, Hangzhou, Zhejiang, China (People’s Republic); The First Affiliated Hospital, School of Medicine, Zhejiang University, Hangzhou, Zhejiang, China (People’s Republic); The First Affiliated Hospital, Zhejiang University School of Medicine, Hangzhou, Zhejiang, China (People’s Republic)

## Abstract

Ultrasound is a promising tool for sarcopenia diagnosis, yet standardized criteria are lacking. We aimed to identify the optimal muscle sites and reference values for diagnosing low muscle mass in Chinese older adults using ultrasound. The study included 1011 participants aged ≥60 years. Dual-energy X-ray absorptiometry (DXA) served as the reference standard for assessing muscle mass. Seven muscle sites were evaluated via ultrasound for muscle thickness (MT) and cross-sectional area (CSA). Our results showed that the biceps brachii CSA (AUC=0.832, 95%CI: 0.793-0.870) in males and tibialis anterior MT (AUC=0.833, 95%CI: 0.789-0.877) in females demonstrated superior predictive power for low muscle mass compared to other assessed parameters. Diagnostic thresholds were < 7.1 cm² for male biceps brachii CSA and < 2.3 cm for female tibialis anterior MT. Ultrasound achieved sensitivities, specificities, and accuracies of 73.9%, 78.6%, and 75.7% in males, and 73.6%, 76.8%, and 75.8% in females, respectively, comparable to bioelectrical impedance analysis (BIA) at 62.6%, 80.2%, and 71.5%. The inter-rater reliability was excellent for biceps brachii CSA (ICC =0.877, 95%CI: 0.677-0.941) and good for tibialis anterior MT (ICC =0.731, 95%CI: 0.610-0.817). Low muscle mass defined by ultrasound correlated significantly with poorer physical performance outcomes, with the effect size higher than DXA or BIA diagnosed low mass, suggesting better prediction power of ultrasound than DXA or BIA. In conclusion, ultrasound could be a preferable method for sarcopenia assessment, demonstrating good inter-rater reliability and significant correlation with physical performance.

